# Bioluminescence imaging to track real-time armadillo promoter activity in live *Drosophila* embryos

**DOI:** 10.1007/s00216-014-8000-8

**Published:** 2014-07-15

**Authors:** Ryutaro Akiyoshi, Taro Kaneuch, Toshiro Aigaki, Hirobumi Suzuki

**Affiliations:** 1 Corporate Research and Development Center, Olympus Corporation, Kuboyama 2-3, Hachioji, Tokyo 192-8512 Japan; 2 Laboratory of Cellular Genetics, Department of Biological Sciences, Tokyo Metropolitan University, Minani-Ohsawa 1-1, Hachioji, Tokyo 192-0397 Japan

**Keywords:** Bioluminescence imaging, *ELuc*, *Armadillo* promoter, Ionomycin, BIO, *Drosophila* embryogenesis

## Abstract

We established a method for bioluminescence imaging (BLI) to track real-time gene expression in live *Drosophila* embryos. We constructed a transgenesis vector containing multiple cloning sites and enhanced green-emitting luciferase (ELuc; Emerald Luc), a brighter and pH-insensitive luciferase for promoter analysis. To evaluate the utility of BLI using an ELuc reporter together with an optimized microscope system, we visualized the expression pattern of *armadillo* (*arm*), a member of the Wnt pathway in *Drosophila*, throughout embryogenesis. We generated transgenic flies carrying the *arm*:: *ELuc* fusion gene, and successfully performed BLI continuously for 22 h in the same embryos. Our study showed, for the first time, that *arm*::*Eluc* expression was dramatically increased in the anterior midgut rudiment, myoblasts of the dorsal/lateral musculature, and the posterior spiracle after stage 13, and the cephalic region at stage 17. To further demonstrate the application of our BLI system, we revealed that *arm* transcriptional activity in embryos was modulated inversely by treatment with ionomycin or 6-bromoindirubin-3-oxime (BIO), an inhibitor and activator of Wnt/β-catenin signaling, respectively. Therefore, our microscopic BLI system is useful for monitoring gene expression in live *Drosophila* embryos, and for investigating regulatory mechanisms by using chemicals and mutations that might affect expression.

**Electronic supplementary material:**

The online version of this article (doi:10.1007/s00216-014-8000-8) contains supplementary material, which is available to authorized users.

**Figure:**
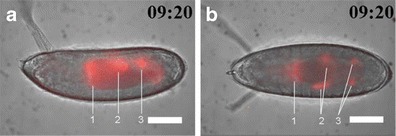
ᅟ

## Introduction

The analysis of gene expression has been widely used as an essential approach to understand complex biological processes. To date, several histological approaches have been employed to better understand the spatiotemporal expression pattern of genes. In addition, live observations have been developed as important techniques to evaluate the effect of genetic manipulations or pharmacological treatments on target gene expression [1–3]. Fluorescent proteins such as GFP from the jellyfish *Aequorea victoria* are applicable for use in live imaging as promoter reporters [4] or as tags for gene products [5]. However, the excitation light required for fluorescence imaging might damage samples owing to phototoxicity, and imaging could be challenging because of autofluorescent backgrounds in live specimens [6–8]. By contrast, the problems associated with fluorescence imaging can be solved using bioluminescence (luciferin-luciferase) imaging (BLI). Although luciferase is widely used as a spatiotemporal reporter of gene expression [9–11], the bioluminescent light emitted from a single cell is very faint. Therefore, an ultra-low-light imaging camera is needed to capture bioluminescent images microscopically [12–15]. We previously developed a microscope with an optimized optical system for BLI using a short focal-length imaging lens to capture brighter images [16].

In addition, the luminescent signal can be increased by using a brighter version of luciferase reporter. Enhanced green-emitting luciferase (*ELuc*; Emerald Luc) is a click beetle luciferase gene that produces >10-fold increased bioluminescence signal compared with firefly luciferase in mammalian cells [17]. Another advantage of *ELuc* is that it is less sensitive to changes in pH; therefore, it is ideal for use in long-term observations [17].

In the present study, we constructed a vector to monitor the expression of a specific gene in *Drosophila melanogaster* by using *ELuc* as a reporter and our bioluminescence microscope developed previously [16]. We focused on the *armadillo* (*arm*) gene, a segment polarity gene whose expression pattern in the early embryo has been analyzed using in situ hybridization previously [18]. However, little is known regarding its expression pattern in later stages of embryogenesis, which involves dynamic organogenesis. Therefore, we analyzed the spatiotemporal expression pattern of *arm* from the fertilized egg to hatching in the same single embryo, which takes ∼22 h, and overlaid the bright field images (BFI) to identify the locations of *arm* in the embryos.

We demonstrated that our method allows real-time tracking of *arm* expression throughout *Drosophila* embryogenesis continuously. Furthermore, we examined the effects of drugs, which have been well characterized in mammals [19, 20], to see how the compounds influence the *arm* expression in *Drosophila* embryos.

## Materials and methods

### Promoter vector

To generate a bioluminescent reporter vector in *D. melanogaster*, we modified the pGreenPelican + attB vector [21]. The GFP-containing *Eag*I fragment of the vector was replaced with a PCR-amplified fragment containing the *ELuc* coding sequence preceded by the *Drosophila* Kozak sequence [22] (pElucPelican + attB, Fig. S1). *ELuc* (Emerald Luc; Toyobo, Osaka, Japan) was PCR amplified using the primers 5′-CGGCGGCCGCCAAAATGGAGAGAGAGAAGAACGT-3′ (forward) and 5′-CGGCGGCCGCTTACAGCTTAGAAGCCTTCT-3′ (reverse). A PCR product (1.8 kb) containing the complete *arm* promoter, including the E16 and E9 first exons [18], was inserted into *Bgl*II and *Eco*RI restriction sites in pElucPelican + attB, to generate a bioluminescent reporter driven by the *arm* promoter (*arm*::*ELuc*, Fig. S1). The *arm* promoter region was amplified from *D. melanogaster* (*y w* strain) genomic DNA using PCR and the primers: 5′-CCAGATCTTCCGCCGCCAGCTGCTGTGACC-3′ (forward) and 5′-CCGAATTCACCACACCTGCAAGAAAGAGACGG-3′ (reverse) [23].

### Transgenic flies

Transgenic flies carrying the *arm*::*ELuc* construct were generated using a phi C31-based integration system [24, 25]. The *arm*:: *ELuc* construct DNA (400 μg/mL) was introduced into the *y* M {vas-int. Dm} ZH-2A *w*; M {3xP3-RFP.attP} ZH-86Fb strain by microinjection using Inject Man NI2 (Eppendorf, Hamburg, Germany). After hatching, luciferase-expressing larvae were selected individually by luminometric observation (Luminescensor; Atto, Tokyo, Japan) after feeding with 100 μM d-luciferin potassium salt (Promega, WI, USA), and the luminous adult males were crossed with virgin *y w* strain females. The red-eyed male progenies were crossed with virgin *y w*; *wg* ^*Sp*-*1*^/*SM1*; *Pr Dr*/*TM6C*, *Sb Tb* strain females to stabilize the *arm*::*ELuc* insertion.

### BLI

Fertilized eggs of the *arm*::*ELuc* transgenic strain were attached to 35-mm glass-bottom dishes using glue and a soft brush and were immersed in 3 mM d-luciferin potassium salt dissolved in Milli-Q (Millipore, Darmstadt, Germany) water for 5 min at 25 °C. In the drug treatment assays, 10 μM ionomycin [19] or 2 μM 6-bromoindirubin-3-oxime (BIO) [20] were added to the solution. After removing the d-luciferin solution with blotting paper, the eggs were immersed in silicon oil (FL-100-1000CS, Shinetsu, Tokyo, Japan) to avoid desiccation and to make the egg chorion transparent.

BLI was performed using a bioluminescence microscope (Luminoview LV200; Olympus, Tokyo, Japan) [16] equipped with a UPlanFl 60× oil objective lens, numerical aperture 1.25 (Olympus), and an electron multiplying charge-coupled device (EM-CCD) camera cooled at −68 °C (iXon; Andor technology, CA, USA). The binning of the EM-CCD was 1 × 1 (512 × 512 pixels), the EM gain was 255, and the exposure times for the bright field (BF) and bioluminescence (BL) images were 100 ms and 4.5 min, respectively, at 5-min intervals for 22 h. The luminescence intensity of the region of interest (ROI) in the embryo was measured using image acquisition and analysis software (Metamorph; Molecular Imaging, CA, USA). Based on microscopic observation of the external embryonic features at 25 °C, the developmental stage was assigned according to Campos-Ortega and Hartenstein’s table [26, 27].

### Fluorescence imaging

To show the autofluorescence of the embryo (*y w* strain), fluorescence imaging was performed using the same system (LV200 microscope, objective lens, EM-CCD camera) used in BLI. In addition, 460–480-nm band-pass excitation and 495–540-nm band-pass emission filters were installed. The EM gain was zero (Normal CCD mode), and the exposure time was 1 s at 5-min intervals for 24 h.

## Results and discussion

### BLI of *arm*::*ELuc* expression during embryogenesis

**Fig. 1:**
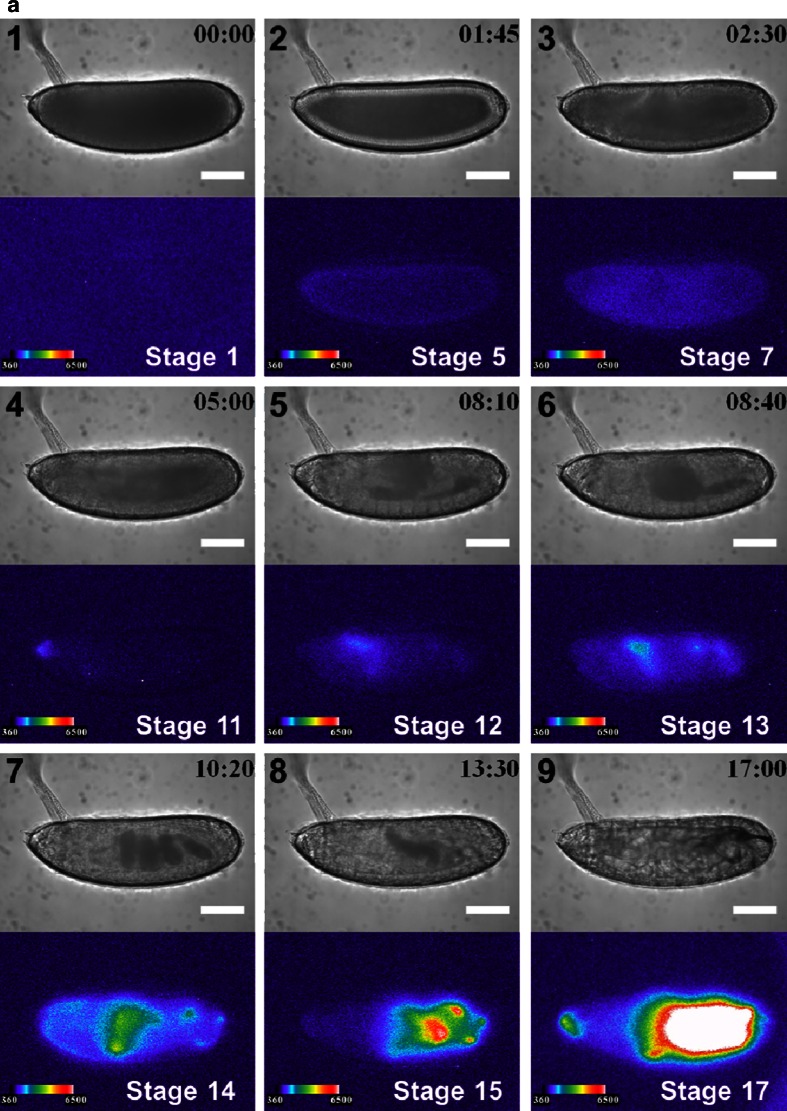

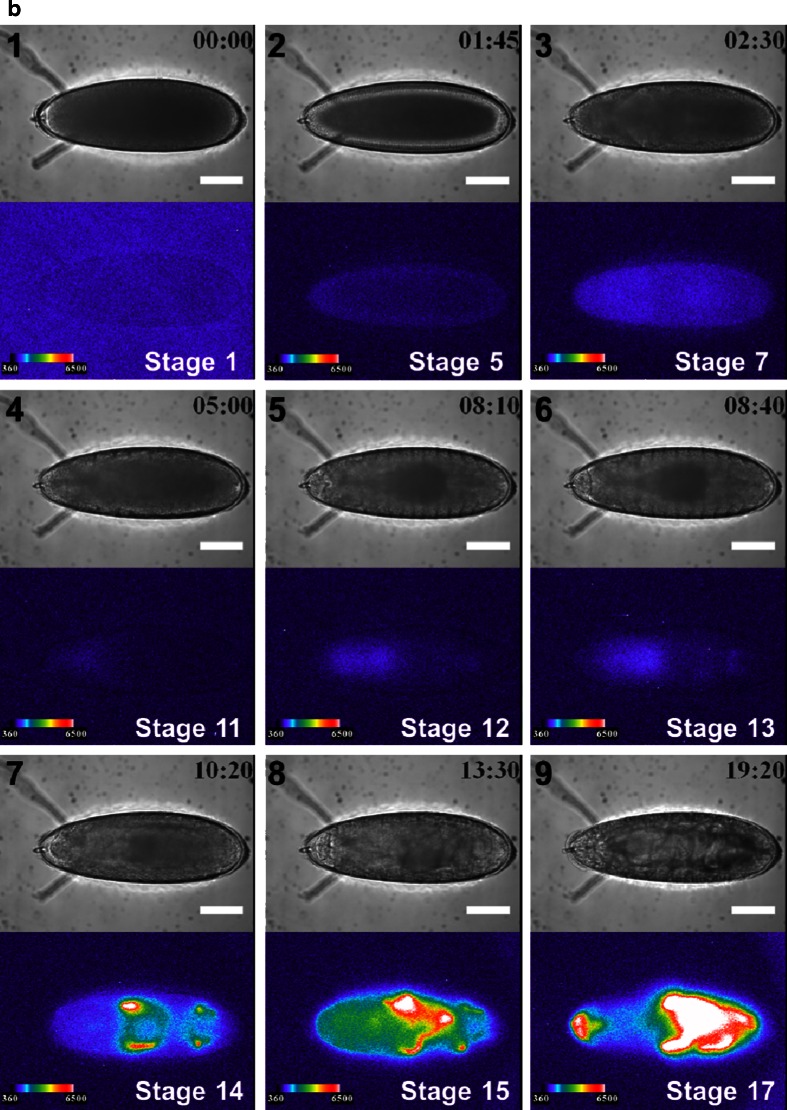
BFI of normal development (*upper panel*) and BL images of *arm* expression (*lower panel*) are shown at several stages with lateral (**a**) and dorsal (**b**) views, with the anterior end to the left. The bright intensity of BLI from 360 to 6,500 of a 16-bit output scale of the CCD camera was assigned rainbow pseudocolors from *violet* to *white. 1*, Stage 1 (0 h after fertilization), no *arm* expression; *2*, stage 5 (1 h 45 min), expression at the cortical cytoplasmic region of the cellular blastoderm; *3*, stage 6 (2 h 30 min), expression in all cell types of the early gastrula; *4*, stage 11 (5 h), expression at 0 % EL; *5*, stage 12 (8 h 10 min), expression in the dorsal side at 75–65 % EL; *6*, stage 13 (8 h 40 min), expression in the dorsal side at 75–65, 35, and 20 % EL. *7*, Stage 15 (10 h 20 min), the expression pattern changed dramatically with morphogenesis; *8*, stage 16 (13 h 30 min), expression moved to 60–0 % EL; *9*, stage 17 (17 h (*lateral view*), 19 h 20 min (*dorsal view*)), expression was maximal at 60–0 % EL, and further appeared in the cephalic region just before hatching. No bioluminescence signal was observed from promoterless control *ELuc* (pELucPelican + attB) construct-inserted embryos (data not shown). *Scale bar* = 100 μm

**Fig. 2:**
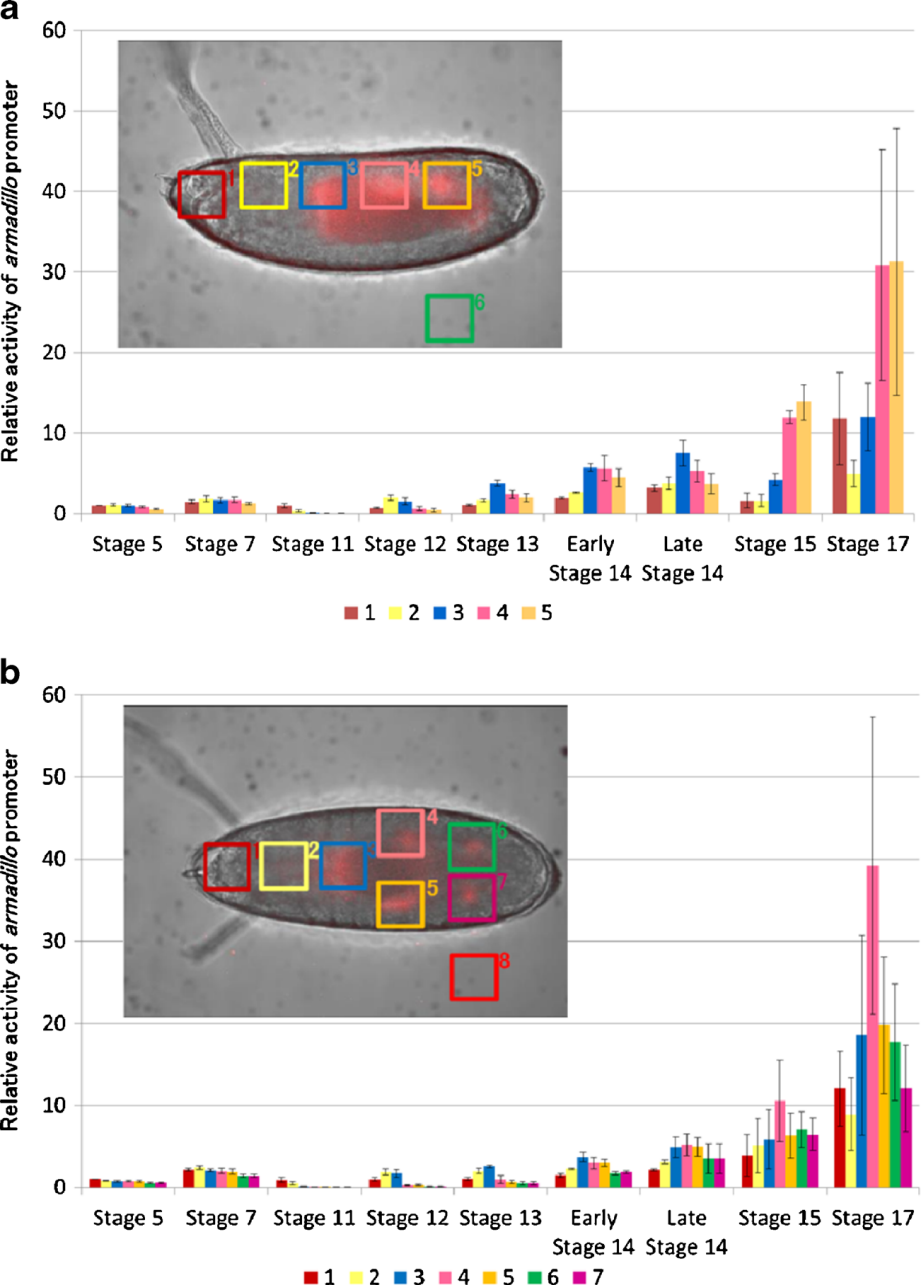
Time course analysis of *arm* expression at six regions of interest (ROI) at several stages with lateral (**a**) and dorsal (**b**) views, with the anterior end to the left. *arm* expression is shown as the luminescent signal ratio of each ROIs per ROI 1 in stage 5 of (**a**) or (**b**) after subtraction of the background obtained from ROI-6 (**a**) or ROI-8 (**b**). Data are presented as mean ± SE (*n* = 3). ROIs were assigned at presumptive areas of the cephalic region (*1*), the proventriculus (*2*), the anterior midgut rudiment region (*3*), myoblasts of the dorsal/lateral musculature region (**a** (*4*), **b** (*4* and *5*)), the posterior spiracle region (**a** (*5*), b), and the background area (**a** (*6*), **b** (*8*))

We performed BLI of *arm*::*ELuc* expression in whole *Drosophila* embryos, which have a high level of autofluorescence, hampering quantitative analysis with a fluorescence imaging. The *arm* gene encodes the Drosophila homologue of β-catenin, a key mediator of Wnt signaling pathway [28], and is also involved in cell–cell adhesion [29]. Thus it plays a critical role during embryogenesis in *Drosophila*. Although *arm* expression has been characterized during the early stages of embryogenesis using mRNA in situ hybridization [18], we attempted BLI of *arm* expression for the entire process of embryogenesis continuously. We constructed an *ELuc*-expressing reporter vector (pElucPelican + attB, Fig. S1), and cloned the promoter region of *arm* into the vector (*arm*::*ELuc*, Fig. S1). *ELuc* expression was analyzed as an indicator of *arm* transcriptional activity. Immediately after fertilization, no *arm* expression was observed (Fig. 1a (1), b (1)). Expression was first detected at stage 5 (1 h and 45 min after fertilization), with weak signals that were likely from the superficial cells of the cellular blastoderm (Fig. 1a (2), b (2)). *arm* expression then spread uniformly over the whole embryo during early gastrulation at stages 6 to 7 (from 2 h 10 min to 3 h 25 min) (Fig. 1a (3), b (3)), and then increased (Fig. 2a, b).

Figure 2 shows the temporal expression profile of *arm* from the lateral and dorsal sides. The ROIs assigned in the embryo were presumptive areas of the cephalic region (Fig. 2a (1), b (1)), the proventriculus (Fig. 2a (2), b (2)), the anterior midgut rudiment region (Fig. 2a (3), b (3)), myoblasts of the dorsal/lateral musculature region (Fig. 2a (4), b (4 and 5)), the posterior spiracle region (Fig. 2a (5), b (6 and 7)), and background area (Fig. 2a (6), b (8)). The *arm* expression disappeared between stages 8 and 10 (3.5–4 h). To exclude the possibility that these expression patterns were produced by nearby enhancers, we generated control transgenic embryos that harbored promoterless *ELuc* (pELucPelican + attB) at the same genomic insertion site. However, no bioluminescence signal was detected in these embryos (data not shown). Therefore, it is likely that the observed expression patterns represent *arm* transcriptional activity.

A previous study showed that *arm* mRNA was expressed abundantly at the preblastoderm stage [18]; however, there was no detectable *ELuc* signal in the current study (Fig. 1a (1), b (1)). This suggests that *arm* mRNA might not be translated at this stage. However, the subsequent spatiotemporal distribution patterns of *ELuc* signal were mostly consistent with the *arm* mRNA expression pattern reported previously [18], suggesting that the *ELuc* expression pattern observed in the current study consistently reflected endogenous *arm* expression.

**Fig. 3:**
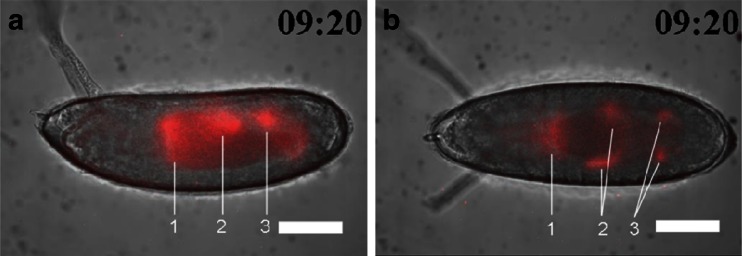
Merged BFI and BL images of *arm* transcriptional activity at stage 14 (9 h 20 min after fertilization) from the lateral (**a**) and dorsal (**b**) side. Increased *arm* expression was observed in the anterior midgut rudiment (*1*), myoblasts of the dorsal/lateral musculature (*2*), and the posterior spiracle (3). *Scale bar* = 100 μm

At stage 11 (4 h 5 min to 5 h 25 min), *arm* expression reappeared at the dorsal region of 100–70 % EL (percent egg length) during the formation of the parasegmental furrow (Fig. 1a (4), b (4)). At stage 12 (5 h 30 min–8 h 10 min), the expression area moved to the dorsal region of 75–65 % EL during germ band shortening and segment formation (Fig. 1a (5), b (5)), and additional expression appeared in two regions at 35 and 20 % EL (stage 13, 8 h 40 min) (Fig. 1a (6), b (6)). These transition processes were confirmed as luminescence intensity in the ROIs from stages 11 to 13 (Fig. 2a, b). Based on the merged BFI and BL images from the lateral and dorsal side at stage 13, the *arm*-positive regions were considered to be in the anterior midgut rudiment, myoblasts of the dorsal/lateral musculature, and the posterior spiracle (Fig. 3). After this stage, the anterior and posterior midgut rudiments elongated longitudinally and fused to become the midgut primordium. The lateral musculature forms muscle fibers to hold the internal organs, and the posterior spiracle forms a tubular structure that connects the tracheal system to the outside of the embryo [26]. Arm protein forms part of the complex that regulates cell growth and cell–cell adhesion [29]. Therefore, the expression pattern changed drastically as morphogenesis proceeded (Figs. 1a (7 to 9), b (7 to 9) and 2a, b; Electronic supplementary material Movie S1). During this period, *arm* expression increased in the 60–0 % EL region (from stage 15, 13 h 30 min to stage 16, 16 h 55 min) (Fig. 1a (8), b (8)). Furthermore, *arm* expression increased dramatically at the cephalic region just before hatching at stage 17 (17 h to 19 h 20 min) (Figs. 1a (9), b (9) and 2a, b), suggesting that *arm* might facilitate the hatching process.

**Fig. 4:**
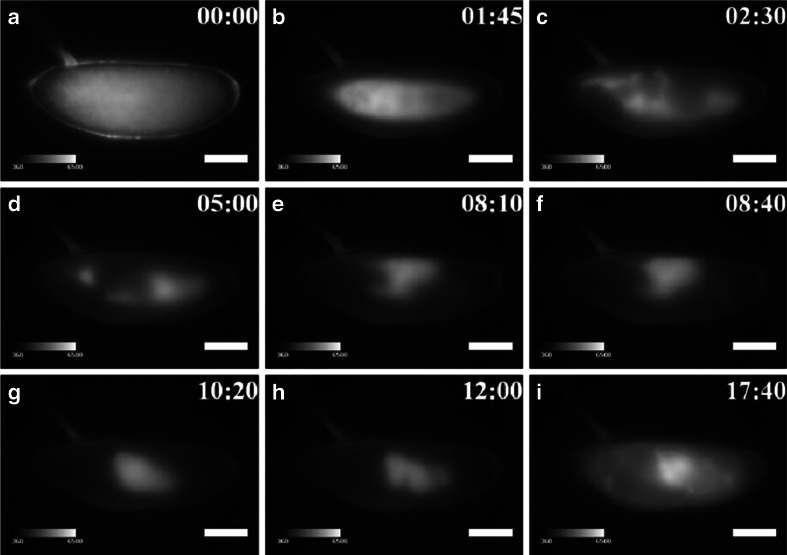
Autofluorescence image of *Drosophila* embryo. Autofluorescence imaging (495–540-nm band-pass) of *Drosophila* embryo excited by blue light (460–480-nm band-pass) shown at several stages with a lateral view and the anterior end to the left. *Scale bar* = 100 μm

Figure 4 shows fluorescent images of embryos excited by blue light (460–480 nm) at several stages from the fertilized egg to hatching using a green-to-yellow (495–540 nm) band-pass filter. Strong autofluorescence was observed throughout embryogenesis, particularly in the yolk and gut system, which would cause problems during the fluorescence imaging of *Drosophila* embryos.

### Effects of ionomycin on *arm*::*Eluc* expression

**Fig. 5:**
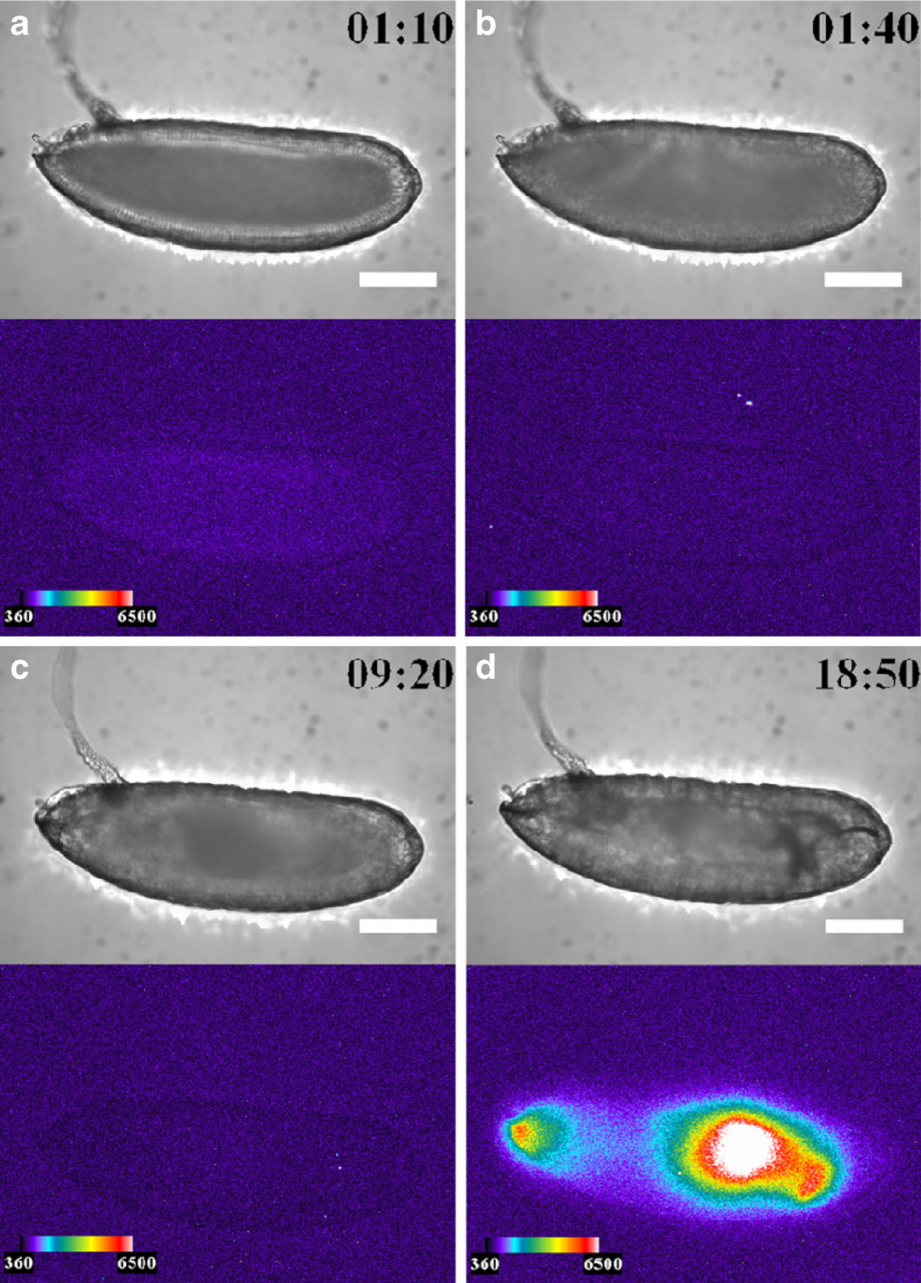
BFI of an embryo treated with ionomycin (*upper panel*) and BL images of *arm* expression (*lower panel*) are shown at several stages with the dorsal side to the top and the anterior end to the left. Bright BLI intensity from 360 to 6,500 of a 16-bit output scale of the CCD camera was assigned rainbow pseudocolors from violet to white. **a** Stage 5 (1 h 10 min after fertilization), *arm* expression at the cortical cytoplasmic region of the cellular blastoderm; **b** stage 6 (1 h 40 min), no expression; **c** stage 14 (9 h 20 min), no expression; **d** stage 17 (18 h 50 min), expression was maximal at 60–0 % EL and further appeared at the cephalic region just before hatching. However, embryonic development was not observed clearly by BFI. *Scale bar* = 100 μm

As an application of our BLI technique, we performed pharmacological manipulations of the Wnt signaling pathway, and observed *arm*::*Eluc* expression during embryogenesis. The pathway regulates the stability and the intracellular localization of Arm protein [28]; however, little is known about the relationship between the Wnt signaling pathway and transcriptional regulation of the *arm* gene. Therefore, we examined whether *arm* expression is altered when embryos were treated with ionomycin, which is known to inhibit β-catenin/transcription factor (TCF) complex formation [19]. Compared with normal development, *arm*::*Eluc* expression levels were low in the cellular blastoderm embryos treated with ionomycin (stage 5, 1 h 10 min, Figs. 5a and 7a). The expression almost disappeared at stage 6 (1 h 40 min, Figs. 5b and 7a) and remained at low levels until stage 15 (13 h 30 min, Fig. 7a), except for a limited increase in ROI-4 at stage 12. These results suggest that the inhibition of Arm/TCF function caused the transcriptional downregulation of *arm*. During this period, intestinal formation could be observed clearly using BFI during normal development but not in embryos treated with ionomycin (Fig. 5c; Electronic supplementary material Movie S2). Expression of *arm*::*Eluc* reappeared at 60 % EL after stage 15, and then increased and spread to the 60–0 % EL region gradually, similar to the pattern observed during normal development (Figs. 5d and 7a (stage 17)). Throughout embryogenesis, *arm* expression was lower in the embryos treated with ionomycin compared with untreated embryos (Fig. 7a). The results suggested that the effects of ionomycin on *arm* expression could be visualized by BLI.

### Effects of 6-bromoindirubin-3-oxime on *arm*::*Eluc* expression

**Fig. 6:**
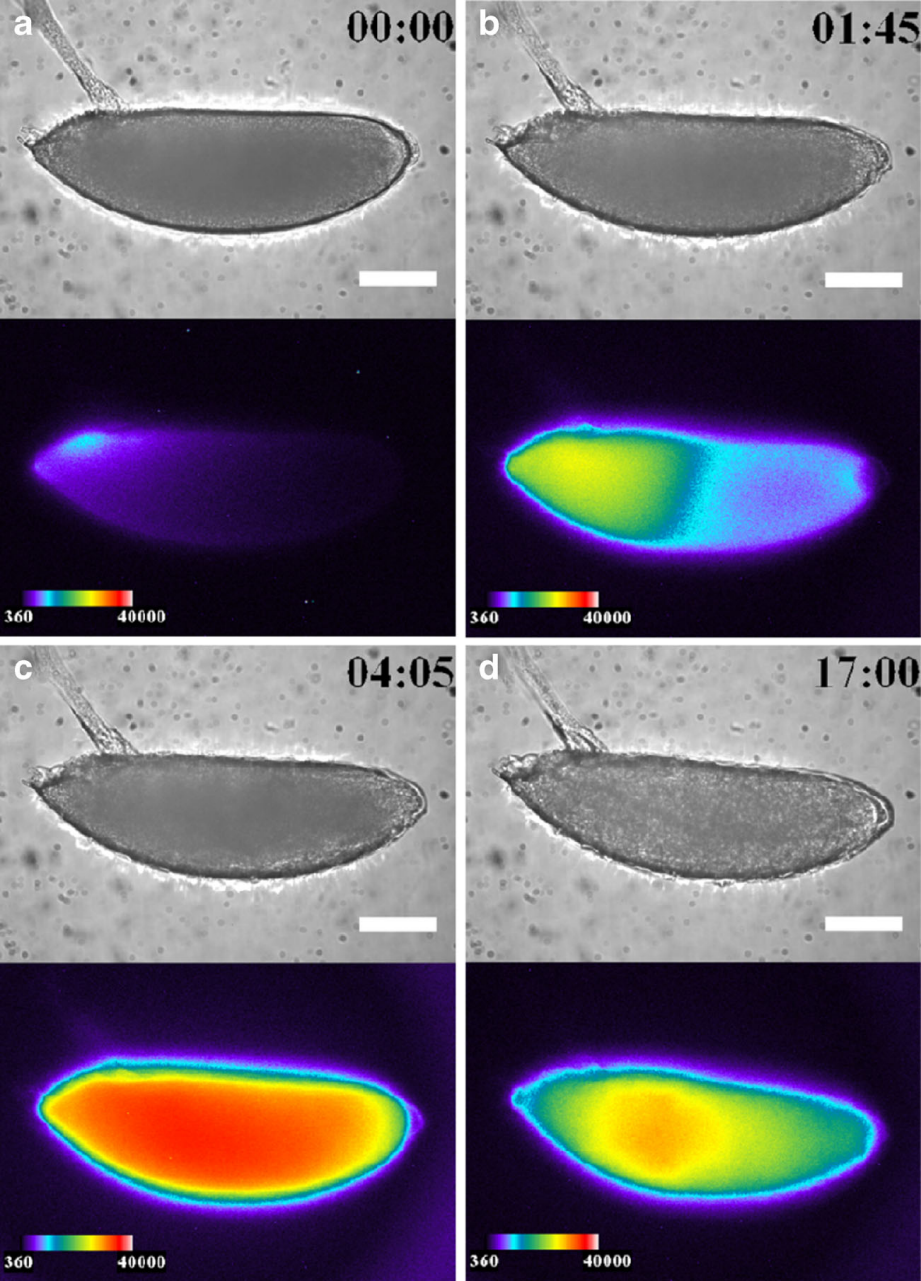
BFI of an embryo treated with 6-bromoindirubin-3-oxime (*upper panel*) and BL images of *arm* expression (*lower panel*) are shown at several stages with the dorsal side to the top of the page and the anterior end to the left. Bright BLI intensity from 360 to 40,000 of a 16-bit output scale of the CCD camera was assigned rainbow pseudocolors from *violet* to *white*. **a** Stage 1 (0 h after fertilization), *arm* expression in the dorsal side at 90 % EL; **b** 1 h 45 min and **c** 4 h 5 min: expression increased and spread over the whole embryo from the anterior to the posterior region; **d** 17 h, expression decreased. Embryonic development was not observed, and the embryo could not hatch. *Scale bar* = 100 μm

**Fig. 7:**
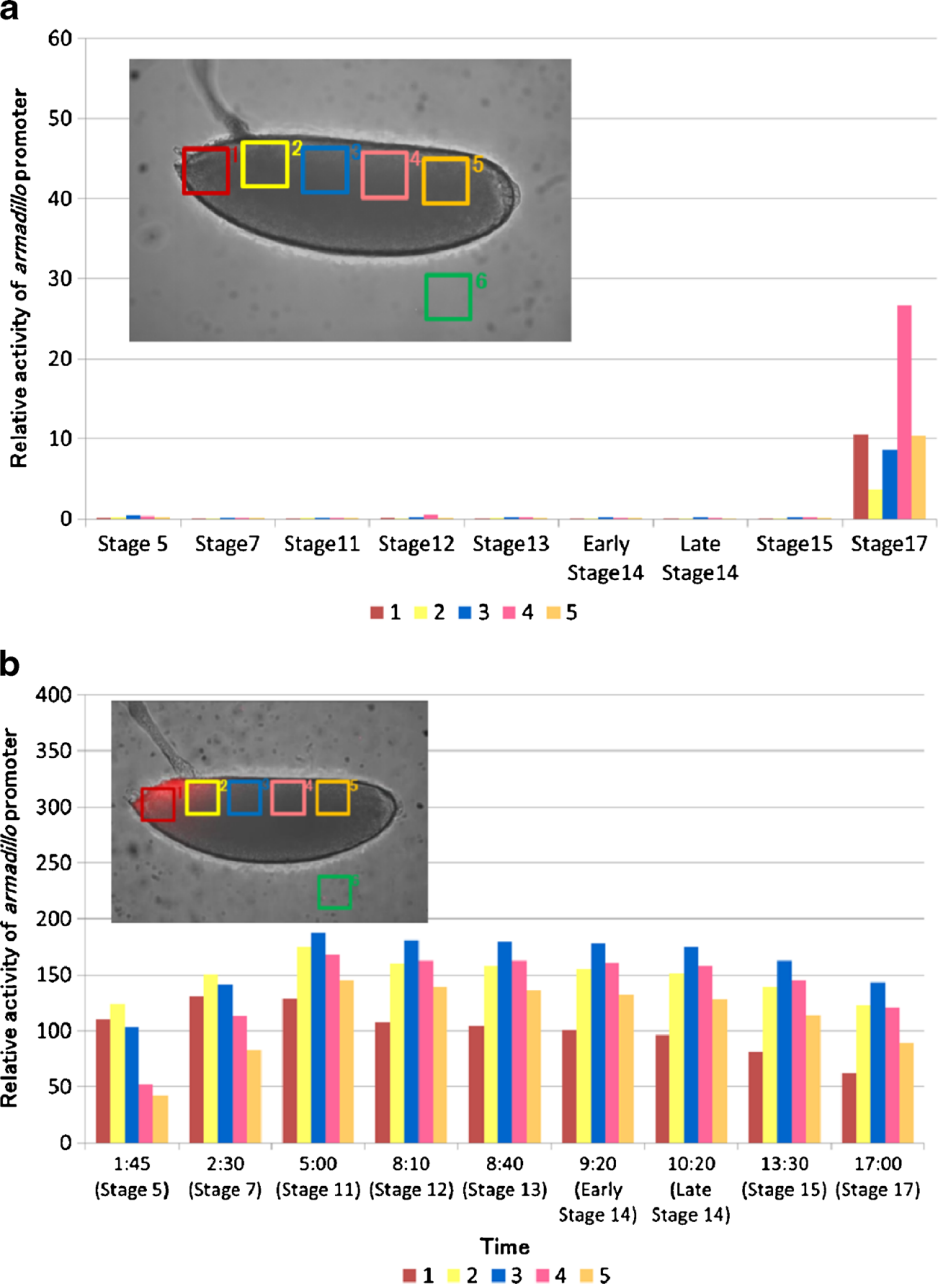
Time course analysis of *arm* promoter activity in six regions of interest (ROI) after treatment with ionomycin (**a**) and BIO (**b**). *arm* expression is presented as the luminescent signal intensity of ROIs relative to ROI-1 in stage 5 of untreated embryos after subtracting the background obtained from ROI-6. ROIs were defined at presumptive areas of the cephalic region (*1*), the proventriculus (*2*), the anterior midgut rudiment region (*3*), myoblasts of the dorsal/lateral musculature region (*4*), the posterior spiracle region (*5*), and the background area (*6*). Because there was no obvious progression in the developmental stages of BIO-treated embryo (**b**), relative intensity of luminescent signal was calculated at the indicated time after egg laying. The embryonic stages in normal development corresponding to each time were shown *in parentheses*

Inhibiting Arm/TCF function using ionomycin seemed to suppress *arm* transcriptional activity. Therefore, we examined the effect of BIO, a glycogen synthase kinase (GSK)-3β inhibitor [20] on *arm*::*ELuc* expression. Because GSK-3β suppresses the nuclear localization of Arm, its inhibition is expected to promote Arm/TCF function. Unlike normal embryogenesis, *arm*::*ELuc* expression was detected at dorsal area at 90 % EL immediately after fertilization (Fig. 6a), and then increased and spread over the whole embryo from the anterior to the posterior region (Fig. 6b, c). We quantified the *arm*:*Eluc* expression level as relative luminescence intensity in the ROIs from 1:45 to 5:00 (Fig. 7b). During this period, no embryonic development was observed using BFI (Electronic supplementary material Movie S3). Finally, the expression gradually decreased from 5:00 to 17:00 (Fig. 7b) and the embryo failed to hatch (Fig. 6d), suggesting that inhibiting GSK-3β impaired embryonic development. Throughout embryogenesis, *arm*::*ELuc* expression level appeared to be increased after BIO treatment (Fig. 7b). These results suggest that *arm* transcription in early embryogenesis might be regulated by the activity of Wnt signaling pathway. However, because these results were described using a single promoter reporter (*arm*) without normalization to an unrelated promoter reporter, the specificity of the effects of these treatments on *arm* expression needs to be confirmed in a future study. Nevertheless, it appears that our BLI system using the *ELuc* reporter is sufficiently sensitive to detect the effects of drugs on *arm* expression during *Drosophila* embryogenesis.

## Conclusions

In the present study, we performed BLI of *arm* gene expression throughout *Drosophila* embryogenesis in the same live embryo, and described the detailed *arm* expression pattern in later embryogenesis for the first time. We also demonstrated the superiority of BLI for *Drosophila* embryogenesis compared with fluorescence imaging, which has challenges that are associated with high levels of an autofluorescent background. In addition, we revealed the *arm*::*ELuc* expression pattern in embryos treated with ionomycin or BIO, an inhibitor and an activator of Wnt/β-catenin signaling, respectively. In summary, we established a BLI method using *ELuc* reporter in *Drosophila*, which could increase the utility of this model organism to study gene expression and regulation in vivo.

## Electronic supplementary material

Below is the link to the electronic supplementary material.

ESM 1 (PDF 117 kb)

ESM 2 (MPG 29.1 MB)

ESM 3 (MPG 9.41 MB)

ESM 4 (MPG 9.90 MB)
