# Supplementary material for: Bioluminescence imaging to track real-time armadillo promoter activity in live Drosophila embryos
Source: Anal Bioanal Chem. 2014 Jul 15;406(23):5703–13. doi: 10.1007/s00216-014-8000-8 (PMC4149885; doi:10.1007/s00216-014-8000-8)
Supplement: Supplementary file 1 — (PDF 117 kb) [file 216_2014_8000_MOESM1_ESM.pdf]

Analytical and Bioanalytical Chemistry

Electronic Supplementary Material

Bioluminescence imaging to track real-time armadillo promoter activity in live Drosophila embryos

Ryutaro Akiyoshi*, Taro Kaneuch, Toshiro Aigaki, Hirobumi Suzuki

Figure S1

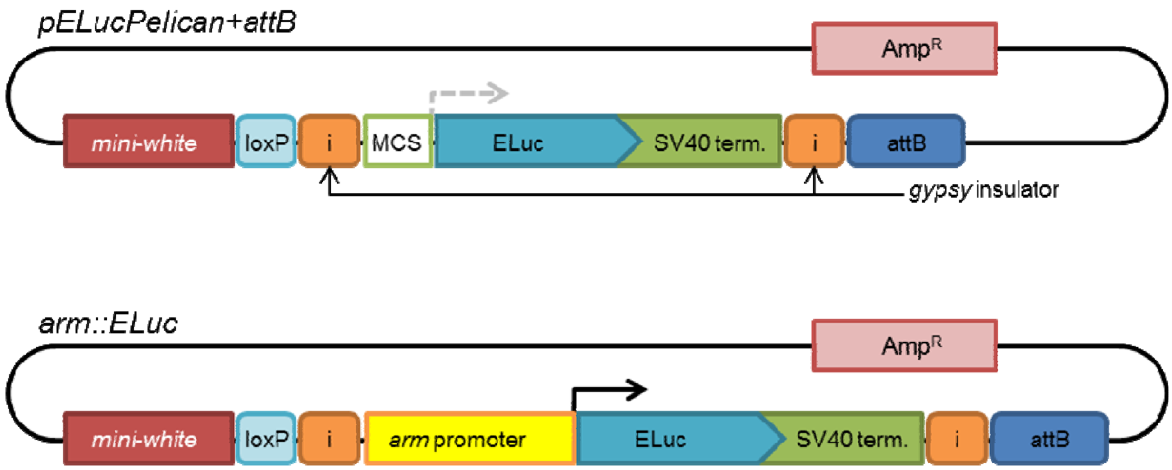

Multimedia files

Movie S1: 216_2014_8000_MOESM2_ESM.mpg

Movie S2: 216_2014_8000_MOESM3_ESM.mpg

Movie S3: 216_2014_8000_MOESM4_ESM.mpg
